# Approximations in English language arts: Scaffolding a shared teaching practice

**DOI:** 10.1016/j.tate.2019.01.004

**Published:** 2019-05

**Authors:** Kristine M. Schutz, Katie A. Danielson, Julie Cohen

**Affiliations:** a University of Illinois at Chicago, 1040 W. Harrison, M/C 147, College of Education, Chicago, IL, 60607, USA; b New York University, 239 Greene Street, Room 634, Steinhardt School of Culture, Education, and Human Development, New York, NY, 10003, USA; c University of Virginia, P.O. Box 400273, Charlottesville, VA, 22904, USA

**Keywords:** Literacy education, Approximations, Practice-based teacher education, Preservice teacher education, Pedagogical tools

## Abstract

Recent research highlights the importance of providing teacher candidates with opportunities to approximate practice. Less attention focuses on tools teacher educators use within and surrounding approximations to focus candidates’ attention on features of practice. This multi-case study investigates how three teacher educators use different approximations in ways that strategically reduce the complexity of learning to teach and scaffold the development of practice. Data indicate teacher educators capitalized on four tools that scaffolded and shaped approximations into spaces for co-constructing shared understandings of practice. These tools include: instructional activities, representations of practice, planning templates, and specified texts and instructional goals.

## Highlights

• Multi-case study of approximations in three ELA methods courses.
• Approximations may serve as a space for developing shared practice.
• Tools used by teacher educators serve as scaffolds for approximations.
• Two new structures and routines for approximations are presented.

## 1 Introduction

Teachers typically improve a lot early in their career (Kraft & Papay, 2014). This suggests there may be lessons learned in the first few years in the classroom that could be potentially traced back to teacher preparation. To better prepare novices who are readier day one, teacher education has recently shifted to focus more on teaching practice, rather than learning educational theory and content knowledge in isolation. Methods courses have become hotbeds of experimentation with different “approximations of practice” (Grossman et al., 2009) such as rehearsals or role plays, which simulate common teaching scenarios and provide opportunities to try out teaching activities with support before using them in real classrooms with real students. These opportunities to practice are designed to “approximate” the situations first year teachers might face as teachers of record,^1^ but with the benefit of concurrent feedback and support from expert teacher educators and peers (Schutz, Grossman, & Shaughnessy, 2018).

While a burgeoning body of literature has explored a particular form of approximation, the “rehearsal of practice,” little research has focused on other forms of approximation that may vary in important ways. Much of the existing research on rehearsals focuses on the structures of the approximations, the roles of candidates and teacher educators (TEs), and the authenticity of the approximation to actual kindergarten - secondary (K-12)^2^ teaching. Less attention has been paid to the tools TEs use both within and surrounding approximations to enable candidates to focus on salient aspects of the practice approximated. If we want maximize the learning potential of varied approximations, we need to build a more robust empirical base about how TEs structure and support candidates as they practice the work of teaching. Tools are important as they have the potential to be used across teacher preparation contexts. TEs in practice-based programs would benefit from guides for support, including resources and materials about facilitating approximations and scaffolding candidate learning in the context of teacher education programs.

As Grossman et al. (2009) noted, if teaching in K-12 classrooms is like kayaking in whitewater rapids with countless variables at play in any moment, an approximation affords TEs the opportunity to “calm the waters” and highlight particular aspects of teaching in a scaffolded way. However, to effectively and strategically calm those waters, we must build more evidence about *how* TEs approach this task and to what effect. Like any learning experience, the approximation alone will not provide the necessary support. The intentional decisions TEs make and the tools they use both surrounding and within an approximation help realize its instructional potential. If we want TEs to be able to carefully scaffold varied approximations of practice across different types of methods courses, we need more research that makes visible the requisite processes, decision-making, and tools.

This paper focuses squarely on these issues, illustrating how three TEs, in the United States, structure three different approximations of practice to strategically reduce the complexity of learning to teach and support the development of high-quality practice. We investigate the tools TEs consider when designing approximations and analyze how these tools provide distinct opportunities for collective learning. Through these illustrative examples, we aim to expand the repertoire of approximations of practice and highlight the critical importance of the TE in facilitating candidate learning during approximations of practice.

We address two research questions: (a) What approximations of practice do English language arts (ELA) TEs use inside methods courses focused on professional practice? (b) What tools within and surrounding these approximations of practice provide a scaffolded experience for learning to teach within a learning community? Using multi-case study, we investigate how three TEs used approximations of practice to support teacher candidates in learning to enact the core practices of facilitating discussion and modeling in ELA, which are at the heart of effective ELA instruction (Duke & Pearson, 2002; Nystrand & Gamoran, 1991; Palincsar & Brown, 1984). These courses are housed in three diverse contexts: an undergraduate literacy methods course situated at an elementary school, a literacy methods course for teachers of record in an accelerated certification program,^3^ and a secondary university-based ELA methods course. The TEs and all authors are members of a cross-institutional consortium engaged in collaborative design and study of core practices for K-12 teaching and TE pedagogies (e.g., rehearsal, TE modeling). This study provides helpful descriptive evidence around tools that contribute to candidates’ scaffolded experiences in learning to teach inside approximations. More broadly, it contributes to a better understanding of forms of approximation that can be used and adapted in varied teacher education contexts and disciplines.

## 2 Background literature and framework

### 2.1 Communities of practice

Sociocultural theory, specifically work on communities of practice, informs the design of this study (Lave & Wenger, 1991; Vygotsky, 1978; Wenger, 1998). We investigate learning as a social process, influenced by interactions among individuals with unique cultural beliefs and attitudes. Group learning in methods courses, specifically the work occurring inside of approximations of practice, functions as a “community of practice.”

Communities of practice have three characteristics: domain, community, and practice (Lave & Wenger, 1991). The domain of interest, in this study is ELA teaching. The community includes individuals building relationships around a joint activity, teaching ELA. Practice accounts for the shared repertoire of experiences, tools, and stories that develop through interactions over time. We draw on these characteristics to understand the communities engaged in approximations. Approximations are the dynamic interaction among multiple factors: participants, tools, texts, physical setting, and the approximation structure. That is, each of these factors contributes to how an approximation unfolds and thus, candidates’ opportunities for learning.

In communities of practice, participants negotiate meaning, allowing members to learn to flexibly apply their learning to new situations. This flexible application involves the use of tools, which may include a written template and shared language. As communities of practice develop, individual participation in the group evolves and understanding deepens, which then influences how tools are appropriated. One tool that may support the development of shared practice is approximations, which are shared publicly with the community of learners and serve as pedagogical tools to support candidates in learning to enact teaching with support.

### 2.2 Approximations of practice

Approximations take myriad forms but are all designed to create a space in which the complexity of learning to teach is reduced to allow teachers to attend to and develop particular aspects of their practice in a safe space with instructional support. Approximations of practice are not simply opportunities to practice teaching. Informed by the theory of deliberate practice (Ericsson, 2002), they are spaces in which candidates work on specific aspects of complex practice. Approximations occur within a community of practice providing candidates the opportunity to appropriate tools from the course and further develop a shared understanding of teaching practice. Inside these spaces, the TE, drawing from multiple bodies of knowledge including an understanding of novice development, shapes opportunities for learning. In approximations, TEs anticipate challenges novices will face in ways that attend to trajectories of learning to teach and “invite novices into certain aspects of practice in order to refine particular elements” (Grossman et al., 2009, p. 2091). Feedback and conversation inside approximations facilitate new understandings of practice and its inextricable link to theory.

Beyond approximation, Grossman and colleagues' (2009) propose two additional dimensions of professional practice: representation and decomposition. Representations are images of practice that make particular aspects of practice visible (e.g., videos, cases). Decomposition unpacks complex aspects of practice making them visible for learners. Within and following the approximations, TEs and candidates decompose practice - naming and describing specific aspects or moves - made visible within the representations created via the approximation. TE-researchers have started to explore the relationship among the three facets of professional practice. For example, in mathematics, Ghousseini and Herbst’s (2016) study of approximations revealed how teaching practice is decomposed and represented inside and across three approximations of practice in a secondary mathematics methods course. Similarly, Schutz and Danielson (2016) found that within rehearsals of read alouds in literacy methods courses, TEs support the decomposition of elements of practice while simultaneously (re)shaping representations of practice made visible to candidates.

Approximations create a space where practice is decomposed and recomposed. Janssen, Grossman, and Westbroek (2015) draw on modularity theory in discussing the need to recompose practice after learning something in isolation. When approximating practice, if candidates only engage in practices at a fine-grained size (e.g., eliciting student thinking vs. facilitating a discussion), it is important to discuss how the piece fits into a larger instructional space (Ball & Forzani, 2009). In approximating practice, different dimensions must be considered, what is being practiced, the context, and the scaffolding for novices (Janssen et al., 2015). The use of modularity theory supports candidates in seeing practice as a whole and in varying grain sizes.

Most research on approximations centers on one particular approximation, rehearsals, where candidates practice teaching and receive in-the-moment feedback when a TE or the candidate pauses teaching. During these pauses, TEs invoke a number of instructional moves to focus candidates on principles, practices, and content. Rehearsals allow for the “deliberate practice of … routine elements as well as opportunities to respond in a principled way to the kinds of non-routine information that comes from students” (Kazemi, Franke, & Lampert, 2009, p. 5). Although initial studies of rehearsal occurred in mathematics education, researchers in multiple domains and disciplines have started to explore the pedagogy of rehearsal (Benedict-Chambers, 2016; Davis et al., 2017). These studies demonstrate how TEs use rehearsal as a space to engage candidates in the complexity of practice, while supporting candidates’ development of pedagogical content knowledge and knowledge of children. Studies of rehearsal across disciplines have shown how TEs utilize varied moves inside rehearsals to address aspects of practice and build knowledge (Barker, 2016; Davis et al., 2017; Kavanagh et al., 2017; Lampert et al., 2013; Schutz & Danielson, 2016).

### 2.3 Scaffolds to support approximations

Researchers are beginning to investigate tools that support approximations of teaching. In approximations, tools scaffold TCs experience as they begin to develop a common language for and vision of practice. Most studies that investigate the use of such tools have examined rehearsals (Benedict-Chambers, 2016; Ghousseini, Beasley, & Lord, 2015; Kazemi, Lampert, & Franke, 2009; Schutz & Danielson, 2016). The literature points to two types of tools used within rehearsals to support approximated teaching: structural tools and interactional tools.

Structural tools scaffold candidates understanding of routines within the practice being approximated. Such tools often serve as common contexts in which TEs support candidates to develop the knowledge, skill, and dispositions for teaching. During rehearsals in mathematics, instructional activities (IAs) are used to structure interactions within the work of authentic problems of practice in teaching (Lampert, Beasley, Ghousseini, Kazemi, & Franke, 2010; Lampert & Graziani, 2009). Janssen et al. (2015), note IAs are a fruitful space to decompose and recompose practice, and protocols have been developed to make this decomposition and recomposition transparent. The use of IAs has also led TEs to develop lesson plans for candidates that highlight particular content and practice, serving as both an additional scaffold and representation of practice. Similar to IAs, TEs have also used frameworks for specific activities to support candidates (e.g., Alston, Danielson, Dutro, & Cartun, 2017; Davis, 2018). Many of these frameworks attend to particular aspects or facets of activities/tasks. For example, science educators utilize a four-element framework to organize science discussions (Davis et al., 2017).

While IAs and frameworks create structures for interaction, other research has examined tools that support candidates’ interactions with students. Interactional tools support participant engagement inside approximations. Ghousseini et al. (2015) investigated the use of a question sequence to support candidates in facilitating interaction during mathematics instruction. This question sequence provided a space inside of rehearsals to engage in adaptive and responsive mathematics teaching. Benedict-Chambers (2016) investigated how specific tools and routines in rehearsals supported candidates in learning about science teaching. One tool supported candidates to interact with student sense-making during science discussions because it previewed potential student misconceptions. The use of questions (Ghousseini et al., 2015) and interactional tools (Benedict-Chambers, 2016) attend to the rich work happening in the approximation. Interactional tools support decisions around what is going on within and surrounding the approximation and bring coherence to the elements that influence the approximation.

Recent research on rehearsals has also attended to how talk can be leveraged as a tool within and following rehearsals to mediate candidates’ understanding. Such research focuses on the particular moves TEs make inside of rehearsals (e.g., Davis et al., 2017; Lampert et al., 2013; Schutz & Danielson, 2016). Lampert et al. (2013) investigated interactions between TEs and candidates during math rehearsals, finding pauses were used to address different structures and substance during rehearsals. Davis and colleagues (2017) found seven purposes for pausing during science rehearsals, the most common being providing feedback to candidates. Schutz and Danielson (2016) examined the moves literacy TEs made during read aloud rehearsals, noting how moves served as critical levers for (re)shaping representations of practice and intervening on pre-existing and sometimes contradicting images of practice. These studies demonstrate the potential opportunity in rehearsals to scaffold candidate learning of in-the-moment instructional decision-making.

## 3 Methodology

### 3.1 Multi-case study

A multi-case study approach was used to gain an in-depth understanding of the approximations of practice TEs used. Case study is an appropriate methodology to investigate the dynamic, multi-faceted nature of approximations as it enables researchers to examine the complex, authentic interactions among participants, tools, and contexts (Merriam, 2009). The participants in this study were three ELA TEs working in practice-focused teacher education programs at public, research universities across the United States. The participants were members of the Core Practice Consortium (CPC), a multi-institutional team of teacher education researchers attempting to develop a collective understanding of core practices and TE pedagogies (Grossman & McDonald, 2008).

### 3.2 Participants and research contexts

**Fig. 1:**
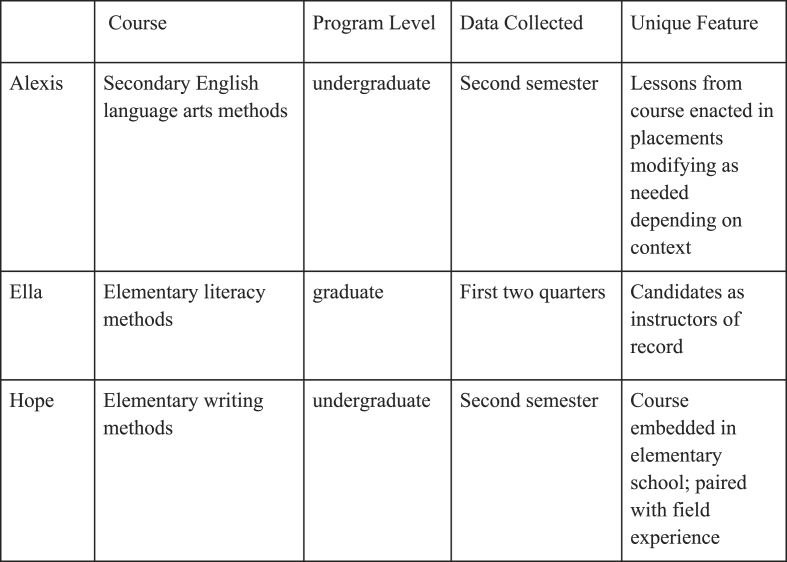
Research contexts.

The TEs had varied levels of experience and taught different courses in different contexts (see Fig. 1). All had been working to develop and document their practice, specifically the use of approximations in their courses.

Alexis^4^ had nine years experience as a TE and taught a secondary ELA methods course during the second semester of an undergraduate program at a university in the Midwest region of America. Alexis's candidates were in field placements during the course, which provided opportunities to enact lessons from the course. One specific lesson required candidates to co-lead discussions with a partner in their placements. Depending on the context, candidates sometimes modified the form of the discussion from what they learned in class. For example, some candidates facilitated discussions with a group of students instead of the whole class. Later, candidates shared video-recordings of these discussions with classmates to reflect on their practice.

Ella had four years of experience and taught a three-quarter elementary literacy methods course sequence in a graduate program at a university in the Western region of America. Candidates were teachers of record while enrolled in the program and enacted what they learned in the course in their own classrooms. Candidates enacted and videotaped lessons in their classrooms as assignments and incorporated IAs and practices as part of their daily classroom routines.

Hope had been a TE for fifteen years and taught a school-embedded writing methods course to first-year undergraduate candidates at another Midwestern university. The course was taught at an elementary school to enable candidates to work with children in classrooms within each class session with TE support. Hope's candidates taught small-group writing lessons each week. Candidates rotated the role of lead teacher weekly, but worked with the same small group of students throughout the course.

### 3.3 Data collection

Data were collected from September to June in 2014–2015. Data included video of course sessions incorporating approximations (N = 6, 2 per site), field notes, four interviews per TE, and course artifacts (e.g., syllabi, lesson plans). Researchers conducted semi-structured interviews using a protocol before and after collecting video data at each site. Initial interviews addressed programmatic factors, course design, and TE's use of core practices and TE pedagogies. All other interviews were designed to allow TEs to preview or debrief observed course sessions to highlight their decision-making.

### 3.4 Data analysis

Data analysis began with inductive and deductive coding using a coding scheme developed by the CPC to capture variations of approximations. The initial coding scheme captured:

● descriptions of the approximation structure, with attention to participant roles, relationship to enactment, and supporting tools
● participant actions and moves

Initial open coding of approximation videos was conducted across the larger research team using Studiocode. After this first round of coding, the CPC research team discussed the coding to build consensus. A sub-group refined the codes for the final codebook.

The first two authors applied the codes to ELA-specific video segments. Videos were initially coded individually, starting with one from each site. Once the initial videos were coded, the first two researchers compared coding. When there was disagreement in codes, the researchers discussed reasoning until reaching consensus. After this initial coding, the ELA team realized a finer-grained analysis was needed to understand specific tools supporting approximation across the courses. Additional codes were developed through discussion, drawing from notes from initial coding. These codes attended to the nature of the tools. For example, it emerged that each ELA-TE considered text selection for approximations – either by selecting a text or constraining text selection. The additional codes captured these TE decisions that connected to the initial coding scheme and may not have arisen with the larger CPC group because they were ELA-specific (See supplementary material for codes).

One video from each site was then watched to refine codes and calibrate reliability. Following video coding, memos were written describing the approximations featured in each TE's practice, the structure of the approximation, participant's roles, and the focus of specific interactions.

Once initial coding of videos was complete, videos were transcribed. Transcription allowed for further analysis of TE language. Transcripts were then uploaded into Dedoose and coded. The same coding scheme was used across videos and transcripts. One researcher compared a portion of video from each site to the coded transcript to check for reliability. TE interviews and course documents were also uploaded and coded in Dedoose. After all documents had been coded, including multiple passes through to look for confirming or disconfirming evidence, data matrices (Miles & Huberman, 1994) and memos focused on the use of tools to support approximations were created for each case. Data were analyzed within and across cases. Case memos were shared with TEs as a way of member checking findings.

## 4 Results

Our analysis revealed three qualitatively different approximations of practice across the methods courses: concentric circles, rehearsals, and work-throughs. The approximations shared several tools that scaffolded candidates’ opportunities to collectively understand and develop teaching practice. Because the core practices TEs targeted within approximations varied, we focus on tools used by all three TEs that were specific to the design of the overall approximations. These tools include: (1) IAs; (2) representations; (3) planning templates; and (4) texts and instructional goals. These tools diminished complexity inside approximations and enabled the approximations to become collaborative spaces for potential learning.

### 4.1 Three approximations of practice

#### 4.1.1 Concentric circles

Alexis strategically used concentric circles to support her secondary ELA candidates in learning to facilitate discussions about texts. Concentric circles were a multi-layered approximation that allowed candidates to practice teaching, act as students, and observe. During concentric circles, candidates were seated in two circles – an inside circle in which candidates either approximated the teaching of a segment of an IA or engaged as fictional students, and an outside circle in which candidates observed and analyzed interactions occurring in the center circle. Throughout the approximation, the TE remained in the inner circle, playing a fictional student. In this role, she made contributions that intentionally probed candidates to use talk moves they had been taught. For example, she responded with a purposeful response to give candidates the opportunity to try “pressing.” She also drew from her knowledge of learners by representing common misconceptions candidates should be prepared to attend to in their teaching. The TE's role remained consistent throughout concentric circles; however, candidates rotated through the roles of teacher, student, and observer. A debrief followed the approximation.

#### 4.1.2 Rehearsals

Ella used rehearsals throughout her elementary literacy methods course to support candidates who were simultaneously teachers of record. The rehearsals were conducted similarly to how they are described in the literature (e.g., Kazemi et al., 2009). One candidate enacted the role of the teacher and simulated teaching a segment of an IA (e.g., interactive read aloud, modeled writing). Other candidates responded as students and observed. Within rehearsal, the TE or rehearsing candidate paused the teaching to coach or discuss aspects of teaching and learning surfaced in the approximation with the group. Then, the rehearsing candidate typically repeated the segment of teaching that was discussed and attempted to apply feedback. Ella highlighted key learnings for candidates’ consideration when planning and enacting lessons after each rehearsal.

#### 4.1.3 Work-throughs with deliberate dives

Hope used work-throughs in her school-embedded writing methods course to support candidates in planning and preparing IAs they then enacted in a classroom. During work-throughs, candidates collaborated in teams, focused on refining a focal candidate's lesson plan throughout the approximation and collaborative planning. Work-throughs occurred simultaneously in multiple teams, and TEs rotated, intermittently joining different teams and engaging in, what we term, deliberate dives. During deliberate dives, the TE observed and extended candidates' contributions or concerns to position the candidates for a successful enactment in classrooms.

### 4.2 Instructional activities

**Fig. 2:**
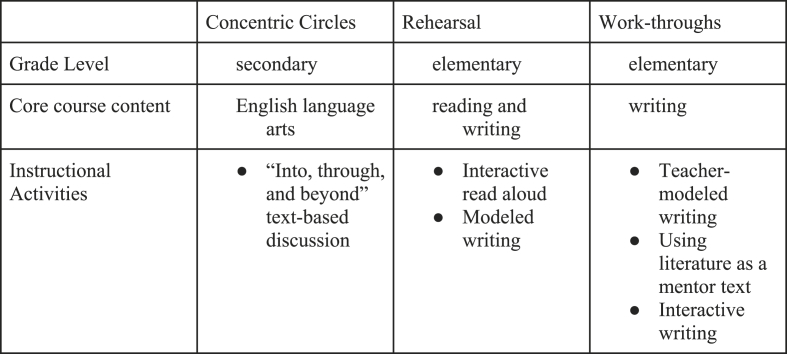
IAs addressed in approximations.

To facilitate approximations of teaching, TEs must supply novices with a specific component of practice to approximate. Across approximations, the TEs designed or selected IAs for this purpose. Although the TEs could have had candidates approximate a single core practice (e.g., eliciting student thinking), instead, all three TEs embedded work on teaching inside IAs. Fig. 2 overviews the focal IAs for each approximation. The IAs differed across approximations and were influenced by course goals and grade level (i.e., elementary or secondary). Across approximations, the IAs served as a “stable and rehearsable backdrop” in which TEs honed in on particular aspects of practice and tightened the range of knowledge of focus (Lampert & Graziani, 2009, p. 493). For example, although there are many structures and purposes for facilitating interactive read alouds, the TE identified *one* that was accessible to novices.

TEs reported using IAs for three purposes. First, the IA served as a common, articulated structure for candidates, thus building a shared understanding of practice. Each of the IAs contained multiple parts and could be segmented to emphasize the different purpose attached to each part. Alexis used the IA of “into, through, and beyond discussion” (Brinton & Holten, 1997), herein referred to as “discussion.” The IA was segmented into three sections; each section had a different purpose and requisite set of teacher moves. For example, in the “into” section of the IA, candidates established norms and prepared students for the discussion using an array of activities to prime background knowledge. These aspects could be flexibly addressed within the “into” section of the IA. Second, the IA served as a space to situate the learning of core practices and specialized content knowledge. Ella described IAs as “a tool to help [candidates] focus in on the content and the core practices.” Hope described how the IA provided a context in which she could talk about practices such as facilitating interaction and modeling, as well as principles of teaching that undergird her course, because in her view of teaching, “everything is so embedded.” Finally, TEs spoke of how the IAs they designed or selected served as portable and “important tools” that candidates could use in the future. Alexis recalled telling candidates, “I'm going to teach you something now that you might use in some kind of permutation, maybe, later on.” Hope stressed how when candidates enter their own classrooms, she wants them to think, “Oh. I can do this because I have this in my toolbox …” referencing the IA.

### 4.3 Representations of practice

TEs used multiple forms of representations as tools to provide candidates with common images of teaching practice. The representations shared with candidates prior to planning for and approximating teaching were created or selected by the TEs to align with their conception of teaching and to make particular aspects of practice visible to novices. The representations took one of three forms: (1) the TE modeled while candidates participated as student learners; (2) the TE shared a video of her teaching the IA in a classroom with children; or (3) the TE shared a video of a novice teacher teaching the IA. Importantly, the representations emphasized aspects of practice the TEs believed were necessary and accessible for novices. This emphasis served as a scaffold to support candidates to notice these aspects; whereas they might not have been as visible in representations that did not attend to novice trajectories of learning to teach. Debrief or discussion about particulars of practice followed the use of these representations. During the debrief, TEs decomposed practice to begin to establish a common and specified understanding of the IA, core practices, and disciplinary content. The representations also often served as common texts or references during the course, with the TE or candidates connecting current learning to the representation previously viewed or experienced together.

#### 4.3.1 Concentric circles

Alexis shared multiple representations of practice to help candidates begin to develop a common vision for facilitating discussion. She recognized that not all candidates had opportunities to observe discussions in the field, and therefore needed to provide in-class representations that highlighted the type of discussion she was preparing candidates to approximate and then facilitate in classrooms. As such, the representations Alexis provided in class through modeling and a video of a novice teacher served as early images for the candidates as they began to develop a shared definition and vision of discussion.

In video records of Alexis' teaching, each representation was followed by a debrief in which Alexis facilitated the decomposition of practice to help candidates name and understand discussion components and instructional moves she deemed critical for novices. For example, after modeling a discussion, Alexis asked candidates to consider the facilitation moves and strategies they had observed her using, and the impact on their responses and learning. The class discussed the experience and their noticings, and Alexis subtly highlighted particular aspects of practice by revoicing candidate contributions using professional language. Following the discussion, she shared prepared slides that provided a clear decomposition of each section of the discussion. For example, she named and discussed three aspects of the “through” part of the discussion as: “(1) using facilitative moves; (2) attending to and assisting student participation; and (3) sharing non-evaluative responses.” She then expanded on each facilitative moves by naming, defining, describing the purpose, and providing examples. Further, within the debrief, Alexis articulated her decision-making for candidates and drew attention to the often invisible intentionality of teacher's real-time decisions. As such, the representation becomes a common image of practice for the community, while the accompanying decomposition and specification of practice support candidates' development of a shared understanding of discussion.

Alexis also shared a video representation of a novice teacher facilitating a discussion. In an interview, she explained this selection, stressing the difference between representations of expert and novice practice. She said, “They are first-year teachers, so it's closer to the kind of work they can do. When I use veteran teachers, [novices] can't see themselves and just don't take away as much.” In this way, she provided candidates with an accessible representation of practice that they could likely relate to and perhaps emulate. The structure of the IA and the facilitation moves Alexis introduced were visible in the video, and some language Alexis had introduced also appeared.

#### 4.3.2 Rehearsals

To support candidates in learning about IAs they would rehearse, Ella both modeled and shared video representations of herself teaching the IAs (e.g., interactive read aloud) to children. She deliberately aligned the representations she created for candidates with the IA and made focal elements of practice visible. For example, when modeling teacher-modeled writing for candidates, she deliberately used marking language such as “Did you notice how I …” to show candidates how to draw children's attention to how she was annotating the writing strategy she had just demonstrated. She made similar, intentional moves in the videos of her teaching, however these videos also showed candidates how children engaged in the lesson.

Debriefs of representations attended to multiple aspects of teaching practice. Ella described how debriefs serve as an integral part of the representations as candidates watch her, and then, together, they “break [the representation] down in all these different ways and talk about what's going on with the content, talk about what's going on with the core practices.” Within the debriefs, Ella also highlighted the instructional decisions she made to help candidates see that instructional decisions are always contextualized and responsive to learners. For example, when debriefing the read aloud she facilitated with children, she helped candidates see and understand specific moves she made to support a child who was reluctant to participate. Although debriefs occupied a lot of time, they served as valuable resources as candidates began to plan for and rehearse the IAs. Ella described how the representations are something the learning community is “constantly coming back to.” She explained how members of the learning community, including herself, prompt the group to recall the model:

We'll be having a discussion or someone will be planning. They'll be wondering about the question to ask. I'll say, *Let's think about when I did [the interactive read aloud of] My Best Friend. What did I do in that moment?* … *W*e're constantly coming back to that, we're breaking it down …

As such, the representations serve as visions of practice to which the community returns, as their own practice develops and experience expands. After being prompted to return to representations to support their own planning or approximation of practice, Ella routinely stressed how candidates needed to “consider the thinking behind moves and how it applies to the candidates’ classroom situation[s].” The goal in sharing these representations is not to encourage mimicry, but rather to help the community begin to develop a shared understanding of practice. This facilitates collective conversation about the moment-to-moment decisions teachers make, and how they impact instruction.

#### 4.3.3 Work-throughs

All representations supporting work-throughs were modeled live by a TE. Hope or her teaching assistant modeled the IA when first introducing it to candidates and co-planned these modeled lessons with another TE to intentionally draw attention to aspects of practice candidates were learning within the IA. Hope described how she “planned really carefully” and “was just very conscious” to make focal aspects of practice like marking and modeling “visible” to candidates. This type of modeling on the part of the TE required extra preparation, both identifying what should be highlighted for candidates in the representation and how it would be done. For example, when preparing to model how teachers facilitate interaction inside of the IA of “using literature as a mentor text,” the TE described how she drafted “a lot of phrases and questions that [she] could use as a bank …” to facilitate talk.

Hope also consistently debriefed representations to help candidates develop a common understanding of teaching practice. For example, she described how they'll “step back,” ask what candidates noticed, then begin to name specific aspects of practice and discuss their importance. One example of this occurred as the TE was helping candidates learn to facilitate conversation among students. After the TE modeled, candidates noted how she coordinated the sharing of student thinking for the group, helping students connect their ideas. The TE then shared specific talk moves and discussed how teachers must shift authority within discussions to help students listen and learn together.

### 4.4 Planning templates

The TEs provided candidates with TE-created planning templates in preparation for approximations. Each planning template was specific to the IA and aligned with the representations of practice TEs shared. Planning templates segmented the activity, provided an extended decomposition of the activity tailored to novice needs, and incorporated professional language previously introduced to candidates. The planning templates served as tools to support candidates’ understanding of the decomposed IA and furthered their shared understanding of practice. Moreover, the segmented structure and professional language provided in the templates supported candidates to begin to develop a common language and more nuanced understanding of the work of teaching located inside the IA.

#### 4.4.1 Concentric circles

Alexis's planning template required candidates to identify two types of learning goals for discussions - “learning for discussion” (i.e., goals specific to ELA-specific discussion practices) and “learning with discussion” (i.e., goals attending to ideas in the text) to help candidates see that discussions can support students' understanding of text and their engagement with disciplinary-specific practices such as citing textual evidence. The planning template included three types of questions a teacher might pose within a discussion: questions that support students to demonstrate knowledge, synthesize knowledge, and grapple with big ideas related to the text. It also prompted candidates to craft initial questions, anticipate possible student responses, and identify possible follow-up questions to press students to elaborate (c.f., Alston et al., 2017). This type of planning template emphasizes that candidates must always consider the students they will teach and think beyond planning initial questions in discussions, as teaching is an interactive, relational endeavor.

The template also served as a tool to help candidates begin to develop a collective framework for eliciting and responding to students. When discussing how she supports candidates’ planning, Alexis emphasized how the template “helps them press, post, and revoice.” The template reinforces professional language to describe talk moves Alexis introduced in a previous class session.

#### 4.4.2 Rehearsals

**Fig. 3:**
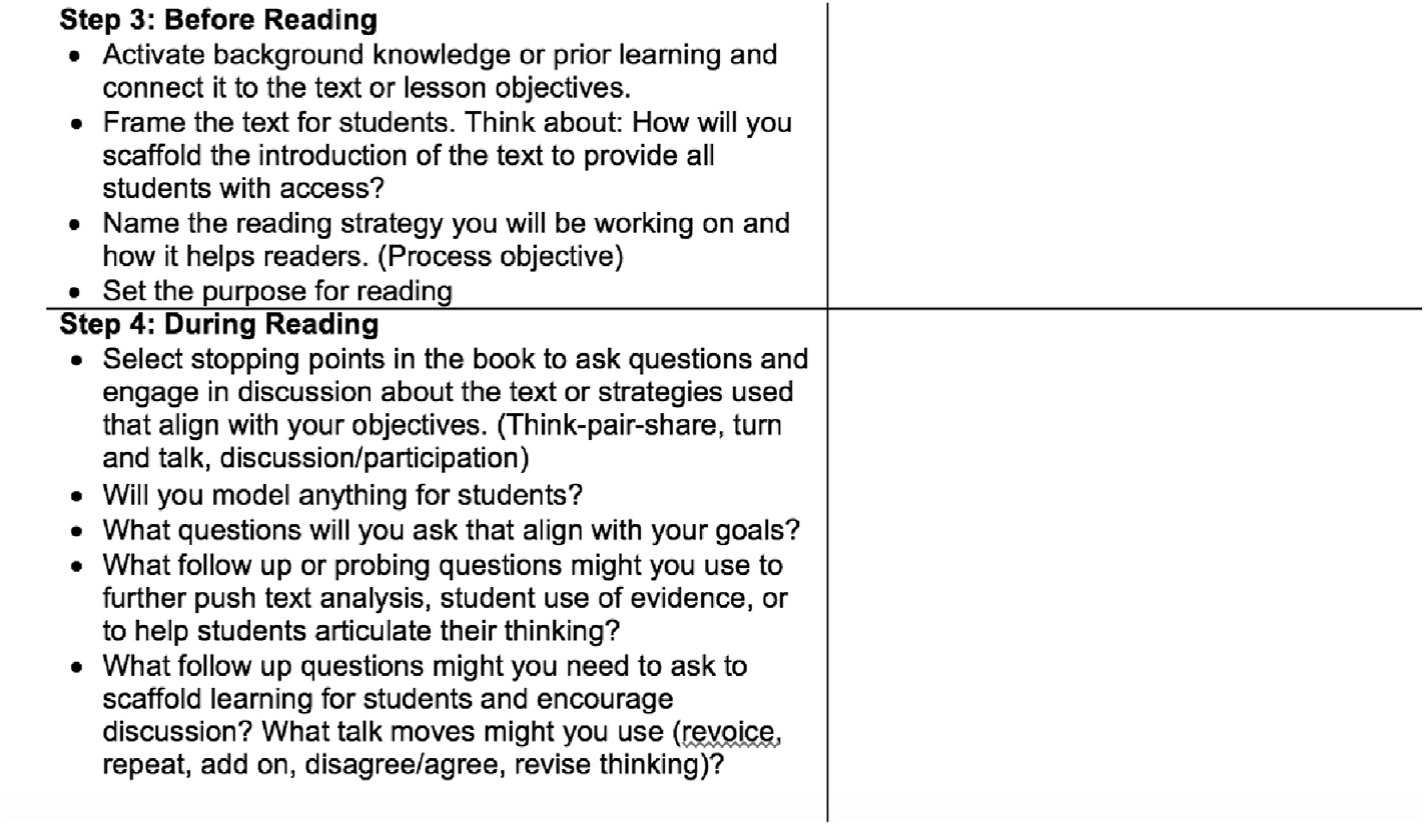
Excerpt for the planning template novices used to prepare for interactive read alouds.

Referring back to the IA Ella previously modeled for the candidates, she described the purpose of the template as, “The IA template decomposes what was just taught for them. It breaks that lesson up into those components.” For example, the planning template for one IA, interactive read aloud, segmented the IA into five steps: text selection and objectives identification, transition to lesson, before reading, during reading, and after reading (c.f., http://tedd.org/activities/interactive-read-aloud/). The planning template further unpacked each step into actionable moves for candidates or questions to consider while planning. In Step 3: During Reading (see Fig. 3), the bullets highlight connecting to prior learning, activating background knowledge, and naming a learning objective. The decomposition of each step in the planning protocol also served as a guide for both candidates and the TE during the rehearsal. For example, prior to her rehearsal, a candidate identified, “… thinking about stopping points and the questions …” as a focal area for her rehearsal. During the rehearsal, the TE paused the approximated teaching and elicited the candidate's thinking about why she had selected specific stopping points. As a group, they discussed the importance of connecting stopping points to learning objectives, linking directly with Step 4 of the planning template (see Fig. 3), and the TE provided an example of how the candidate could phrase a question to do so. Finally, the template served as tool around which candidates could begin to acquire professional terms to describe teaching or content within their community. For example, the template specified how interactive read alouds need content and process objectives. In multiple rehearsals, candidates shared these objectives before rehearsing and incorporated the language into moments in which they were discussing their teaching with the group.

#### 4.4.3 Work-throughs

Hope's template for modeled writing segmented the IA into multiple segments and further unpacked each segment. In addition to listing constituent elements in each segment, she also provided sample language for candidates to use to script their lessons. To help candidates develop teacher language to support children in activating background knowledge, she provided the following example in the template, “Example: I remember last week X used a question to start her story. That was a great strategy. I want to think about another strategy for writing strong beginnings that hook the reader. One thing great authors sometimes use is dialogue. Raise your hand if you could share what the word ‘dialogue’ means. Build on child's response.” The template offered a model of concise and economical language. Hope described the IA-specific lesson templates as “very detailed,” but stressed how candidates “kind of customize it and work with it.” Inside work-throughs, candidates frequently used planning templates as tools as they began to develop a common understanding in their teams. For example, the transcript below shows how one candidate appropriated language from the template in her work-through:

**Table:** 

Approximating Candidate:	Okay, so we're doing another mini lesson this week. Who can tell me what we focused on last week? What was our main goal?
TC 1:	The hook of a personal narrative?
Approximating Candidate:	The hook. Exactly. And what type of hook did we use?
TC 2:	Questions
Approximating Candidate:	A question, yeah. This week, we're gonna work a different type of hook and we're gonna use dialogue. Does anyone know what that word means? Dialogue?

The approximating candidate in this example draws on the language provided by Hope in the planning template, but rather than making a statement about what students have and will learn, she uses questions to address the same information. Following this moment, her team discussed the teaching, concluding through collaborative discussion that it might be helpful for her to summarize the instructional purpose of the lesson for emphasis after facilitating this conversation with students. Because the TE was not always present in work-throughs, candidates depended on the template to serve as not just a guide for planning, but also a guide for providing feedback to peers during the approximation.

### 4.5 Texts and instructional goals

Across the three approximations, TEs prescribed or held some combination of the instructional text and instructional goals constant to hone the focus of the approximation. These constraints were often designed in response to the contexts in which the approximations were situated. All TEs held both the text and instructional goal constant for planning and the approximation to some extent. Identifying the text and instructional goal constrained the focus of the content during the approximations and has the potential to give candidates experiences interacting with the limited content in deep ways within and surrounding the approximation.

#### 4.5.1 Text

##### 4.5.1.1 Concentric circles

Alexis purposefully selected the text candidates used in concentric circles. All candidates analyzed and planned to approximate facilitating discussion of segments of the same text, one that she had previously used to model facilitating a discussion with the candidates. As such, candidates considered the text prior to engaging in concentric circles in each of the three candidate roles (i.e., approximating teacher, fictional student, observer). This influenced the approximation in two ways. First, it enabled the fictional students to respond without hesitation when the teacher posed questions. Alexis explained how this created a dynamic different than what candidates would likely experience in classrooms. She discussed how because the English majors in her class have strong comprehension and are “so adept at participating in a discussion,” how they enacted their roles as fictional students during concentric circles was “not what they will then be involved in a classroom.” She stressed how they'll need to learn to “manage the five [students] up front who always talk with the ten in back who never talk” or the “one student [who] says something that you're not sure connects.” Since candidates often struggle to manage student engagement, their automatic participation decreased the complexity of the activity and allowed the approximating teacher to focus on aspects of the IA that Alexis had emphasized in the specification. Second, the deep knowledge of the text across all participant roles situated the fictional students and observers inside the teaching in a way that positioned them to learn from it across the roles of teacher, student or observer.

##### 4.5.1.2 Rehearsals

During rehearsals, the TE controlled the text selection for the first cycle of an IA, but allowed candidates to select texts in subsequent cycles to enable connections to the school curriculum and their students. The candidates planned for, rehearsed and enacted an interactive read aloud of the same text that Ella had shared a video of herself teaching. In the next cycle, candidates selected their own texts. In some ways, this changed the nature of Ella's role during rehearsals. She described the impact of the “text factor”:

I haven't necessarily read every book. There's some in-the-moment decisions that I'm also making as a teacher educator because I can't anticipate everything that might come up.

When TEs are familiar with a text, this enables them to anticipate resources and challenges of the text, then support candidates in leveraging or attending to these in rehearsals. However, without this knowledge, the TE's role in the learning community shifts; the TE experiences the rehearsal similarly to how candidates do. In rehearsals where Ella was unfamiliar with the text, she often initiated pauses saying, “As a learner, I'm wondering …” or “As a learner, I'm feeling …” whereas she did not use this phrase when she knew the text.

##### 4.5.1.3 Work-throughs

Because work-throughs were used in a writing methods course, the TE controlled the text selection differently. When candidates were focused on the IA of literature as a mentor text, Hope modeled using the mentor text *A Chair for My Mother* by Vera B. Williams. Candidates then used this same text in work-throughs. Keeping the text consistent enabled candidates to see how an experienced TE would use the text inside the IA and presented an image of how instruction could potentially unfold. In a different IA, teacher-modeled writing, candidates used self-authored personal narrative texts. The self-authored texts served as starting points when candidates developed their plans for teacher-modeled writing, as the IA requires teachers to demonstrate and explain how to use writing strategies in their *own* writing. Although the text was not then prescribed, the TE could ensure that the instructional texts (i.e., the self-authored narratives) had a common set of features (e.g., sequenced events, conflict).

#### 4.5.2 Instructional goal(s)

**Fig. 4:**
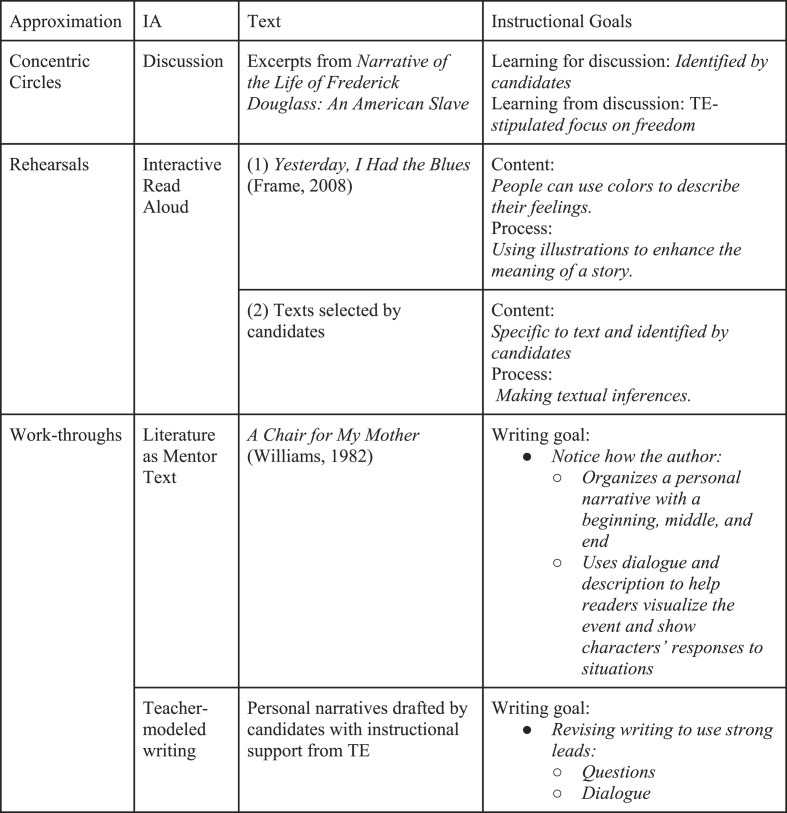
Examples of text selection and instructional goals.

TEs constrained the instructional goals in two ways, by providing broad guidance and parameters or explicitly stating the goal for candidates. Fig. 4 exemplifies how TEs varied approaches to stipulating instructional goals for approximated teaching. Teaching reading and writing requires considerable content knowledge. Constraining the instructional focus allowed candidates to begin to develop a deeper understanding of some key literacy content (e.g., text analysis, genre knowledge, cognitive reading strategies).

##### 4.5.2.1 Concentric circles

Alexis constrained the instructional goal by articulating a theme for candidates. Because their discussions occurred in different contexts, she had candidates identify learning goals related to the theme for the students in their placements. Specifically, she asked candidates to focus on “issues of freedom” raised inside the common text; within that theme, candidates identified their own goals when planning for concentric circles. This TE decision ensured candidate goals connected to the text but provided flexibility for candidates to consider the students they teach when creating the lesson objective. This intentional decision likely highlighted for candidates the importance of considering the specific students in a classroom when planning for discussions.

##### 4.5.2.2 Rehearsals

For rehearsals, Ella fully articulated the instructional goals for the first set of rehearsals of interactive read alouds and partially articulated them for the subsequent set of rehearsals. That is, when all candidates prepared to teach using a common lesson plan she had designed for a common text, both the instructional goals focused on content and process were identified. Ella rationalized this decision explaining that from her past experiences, she knows that candidates need more support in the form of lesson plans and instructional goals, “*because they don't yet have the content knowledge to successfully plan* … *and they struggle to plan without enough knowledge.*” As candidates deepened their understanding of texts and the reading process, Ella shifted her approach for the second round of rehearsals. She still articulated one aspect of the instructional goal, the process goal, as making textual inferences because candidates often need additional support understanding this strategy and explaining it to children. But, candidates identified the content goal for the interactive read aloud to align with their self-selected texts.

By constraining the focus of the lessons, the TEs created a space where the group of teacher candidates could begin to interrogate and understand the content more deeply together. For example, in many rehearsals, the TE was able to support candidates in developing child-friendly explanations of inference, and the group was exposed to multiple applications of inferring across a variety of texts. Thus, the common instructional goals engaged non-rehearsing candidates in the rehearsals, thus strengthening the shared community, because they too needed to deepen their knowledge for their own upcoming teaching experiences.

##### 4.5.2.3 Work-throughs

In preparation for work-throughs, candidates co-planned lessons focused on the instructional goals that Hope identified each week. For example, planned writing mini-lessons focused on using dialogue to create strong leads in personal narratives. These instructional goals were linked to fundamental content knowledge for teaching writing and provided candidates with opportunities to begin to co-construct understanding within the work-throughs. Further, although only one candidate in each work-through team approximated teaching, all candidates enacted teaching to some extent following the approximation, either as the lead teacher in a small group, or in one-on-one conferences. As such, the common instructional goal likely helped create a shared focus for candidates during the approximations.

## 5 Discussion and implications

This study examined the approximations used in ELA methods courses and corresponding tools that provide candidates with scaffolded experiences for learning to teach. Findings from this study expand our understanding of approximations of practice in three ways. This paper presents two approximations of practice-- concentric circles and work-throughs—heretofore unrepresented in teacher education research. The findings also expand the suite of tools TEs should consider as they design approximations. The scaffolds surrounding the approximations - IAs, representations of practice, planning templates, specified texts and instructional goals - transform the experience into a space in which teacher candidates begin to construct a shared language for describing teaching and a common vision of teaching practice. These tools do not appear to operate independently of one another. Rather, the TEs, who facilitated the approximations, coordinated the set of tools to align with a coherent vision of instruction they aspire to communicate to teacher candidates.

These findings augment the existing body of literature on tools that serve as structures and routines for approximations of practice. The TEs all situated teaching inside of IAs. These IAs served as predictable and manageable structures in which instructional routines were situated (Lampert & Graziani, 2009). Across all three courses, IAs were not only used to bound the work of teacher candidates during the approximation, but they also served as structured spaces for addressing content and practice (Lampert & Graziani, 2009). Further, the use of IAs to organize and scaffold work within and surrounding the approximation supported TEs in engaging candidates in multiple decompositions and recompositions of practice (Janssen et al., 2015).

Our analysis shows how TEs deliberately aligned structural and interactional tools that decomposed and represented practice to support approximations. The idea that these components of professional practice interact in a symbiotic fashion is not new. Approximations, decompositions, and representations of practice frequently overlap in professional preparation (Grossman et al., 2009). However, our findings indicate that the TEs’ pedagogy in the study was nuanced and deliberate, with the goal of explicitly connecting the tools they designed and approximations they used to support teacher candidate learning. This finding appears to be in line with the work of Ghousseini and Herbst (2016) who stress how “teacher educators must enact these pedagogies deliberately, to attend to knowledge, practices, a vision of teaching, and a set of productive dispositions.” (p. 101) Across approximations, the TEs designed tools to provide consistent messaging and images about the IA of focus. There was alignment across the IA, planning templates, and representations of practice.

The deliberate work by the three TEs in this study began with the controlled representations of practice they shared. Rather than sharing videos of expert teachers, the TEs created representations practice, either by modeling live with candidates or sharing recordings of their own teaching with students. Each of these representations was planned and created with novice learning in mind. This ensured that representations aligned with the decomposition of practice imbued via the planning templates and debriefs facilitated by the TE. The coherent messaging enables approximations to become fertile ground where candidates experiment with the interactional work of teaching as they co-construct a shared vision of and language for practice. This allows candidates to develop a common foundation for teaching from which they can then learn to improvise. Without this kind of coherence, candidates might focus their energy on managing the cognitive dissonance associated with misaligned tools.

Planning templates and debriefs also appeared to shape the shared experience of the candidates. TEs made strategic choices to limit the variation of instructional texts and goals within approximations. The TE representations, including the text and instructional goals, often mirrored those candidates used during approximations. Teaching English language arts requires deep content knowledge, and holding the text and instructional goals constant enables TEs to support the candidates in acquiring specialized content knowledge necessary for specific instructional goals. By opting for depth over breadth of knowledge in early methods courses, candidates come to understand what it means to know content deeply and the critical role it plays in the relational work of teaching. Moreover, because the content is focused, the work inside of approximations can attend to teaching practices and the relationship to this content thus strengthening the community's collective understanding of the desired outcome.

Although all the approximations provide opportunities to develop interactive aspects of instruction, they each support distinct, additional goals. Work-throughs provide a space for candidates to co-construct understanding, as they toggle between approximating collaborative lesson planning and interactive teaching. In contrast, rehearsals focus entirely on those interactive aspects and situate feedback inside the approximation. The pauses within rehearsals are public, collaborative representations of the inner-thoughts practicing teachers have as they encounter pedagogical dilemmas during instruction. Finally, concentric circles approximate both interactive teaching and peer observation. Within the approximation, candidates experience teaching from three different perspectives – teacher, student, and observer – thus allowing candidates to see the same component of practice from multiple lenses. Given the multiple roles teachers play in schools of today, it is critical that TEs not only support candidates in learning to enact teaching, but also design experiences where candidates are supported in learning with and from colleagues. Collaborative planning and peer observation are aspects of practice critical to fostering teachers’ professional growth (Darling-Hammond & Richardson, 2009).

This study sheds light on the ways in which approximations of practice are spaces where TEs may temporarily reduce the complexity of teaching in order to scaffold candidate learning. As TEs grapple with incorporating different approximations into their methods courses, it is essential that they have varied “images of the possible”, coupled with an understanding of the tools needed to actualize the learning potential of those approximations. These findings are helpful on both fronts. We demonstrate that approximations can take myriad forms beyond the oft-documented “rehearsal of practice.” TEs can and should customize the structure of the approximation, in response to learning goals and context. We also demonstrate the ways in which decision-making regarding associated tools might potentially enhance the learning experiences for candidates. Building a more robust understanding of a suite of tools to support approximations is essential to enhancing the quality of rigorous practice-based teacher education.

## 6 Limitations

While this study makes important contributions to the field of teacher education, it is not without limitations. All researchers and participants were members of the CPC at the time of the study. As such, the CPC community likely shaped the TE pedagogies, by design. One goal of the consortium was to share and refine TE pedagogy, and the features of the approximations we observed were informed by our own shared understanding of practice, which evolved over many years of collaboration. These TEs and the programs in which they work are not designed to be representative of teacher education programs or TEs nationally. That said, these cases are designed to demonstrate variability in the use of approximations, in the context of longstanding collaborative work of the consortium.

## 7 Conclusion

Teaching is complex, relational work that requires rapid decision-making as teachers face pedagogical dilemmas in real time. Ultimately, TEs strive to help candidates develop adaptive expertise that will inform the decision-making and interactions inside everyday instruction. Whether candidates are encountering the “blooming buzz” of classroom life (Brown, 1992) or the “rapids of real practice” (Grossman et al., 2009, p. 2007), the contingent factors that classroom teachers face on a daily basis can overwhelm novices. As such, scaffolds that diminish the complexity of teaching are necessary to support novices in learning to teach. Approximations of practice are a promising approach to quieting the buzz and calming the rapids. But, approximations *can* involve real students, the most unpredictable factor in the mind of a novice. Approximations require structures and tools to support candidates in learning to teach in a principled and deliberate way.

## Funding

This collective work has been supported by the Bill & Melinda Gates Foundation under Grant #OPP1089179 and the Spencer Foundation under Grant #201600110. Any opinions, findings, and conclusions or recommendations expressed in this material are those of the author(s) and do not necessarily reflect the views of the funders.

## Footnotes

1 American term describing practicing teachers.

2 Ages 5–18 years old.

3 The second author was the teacher educator in this setting.

4 Participant names are pseudonyms.
