# Supplementary material for: Approximations in English language arts: Scaffolding a shared teaching practice
Source: Teach Teach Educ. 2019 May;81:100–11. doi: 10.1016/j.tate.2019.01.004 (PMC6472320; doi:10.1016/j.tate.2019.01.004)
Supplement: Multimedia component 1 [file mmc1.docx]

**Supplementary Material**

*Structural Tool-Focused ELA codes*

| **Instructional Activity** | *What instructional activity are TCs approximating?* | |
| --- | --- | --- |
|  | **Interactive Read Aloud** | |
|  | **Modeled Writing** | |
|  | **Using Literature as a Mentor Text** | |
|  | **Discussion** | |
|  | **Small Group Reading Instruction** | |
| **Planning** | *What tools are provided to TCs to support their planning?* | |
|  | **Detailed Planning Protocol** | TC uses a template that is activity-specific and provides guidance about what to say and do within the activity. |
|  | **Specific Template** | TC uses a template that is activity-specific but does not provide guidance as to what to say or do. Instead, it contains broader activity-specific headings or sections. |
|  | **General Template** | TC uses a template that is activity-generic and doesn’t provide guidance about what to say and do within the activity . |
|  | **No Template or Protocol** | TC plans a lesson absent a specific protocol or template. |
| **Representations** | *What tools are used to represent practice?* | |
|  | **TE modeling** | TE models the IA or practice with candidates playing the role of students. |
|  | **TE-created videos** | TE creates a video of herself teaching the IA or practice in a classroom with students. |
|  | **Novice teacher videos** | TE shares a video of a novice teacher teaching the IA or practice in a classroom with students. |
|  | **Expert videos** | TE shares a video of an expert teacher teaching the IA or practice in a classroom with students. |
| **Instructional Goals** | **TE fully constrains** | TE articulates the instructional goals for lessons. |
|  | **TE partially constrains** | TE stipulates some aspect of the instructional goal(s) for approximations or classroom teaching. For example, all candidates must write lessons about making inferences, or all candidates must address issues of bias in their instructional goals. |
|  | **No constraints** | TCs write lessons to approximate or enact in classrooms without instructional goal constraints. |
| **Text** | *Who selects the text for the lesson?* | |
|  | **TE** | TE selects text |
|  | **TC with guidance from TE** | TC selects text with guidance from TC. For example, the text must be an informational text, or the text can be selected from a particular anthology. |
|  | **TC without support from TE** | TC selects text without support from TE. For example, TC selects text based on cooperating teacher request or own interest. |
|  |  |  |
